# Hyperhomocysteinaemia is an independent risk factor of abdominal aortic aneurysm in a Chinese Han population

**DOI:** 10.1038/srep17966

**Published:** 2016-02-11

**Authors:** Jie Liu, Shang Wei Zuo, Yue Li, Xin Jia, Sen Hao Jia, Tao Zhang, Yu Xiang Song, Ying Qi Wei, Jiang Xiong, Yong Hua Hu, Wei Guo

**Affiliations:** 1 Department of Vascular and Endovascular Surgery, Chinese PLA General Hospital, Beijing 100853, China; 2 Department of Vascular Surgery, Peking University People’s Hospital. Beijing 100044, China; 3 Department of Epidemiology and Biostatistics, School of Public Health, Peking University Health Science Center, Beijing 100191, China

## Abstract

The associations between hyperhomocysteinaemia (HHcy), methylenetetrahydrofolate reductase (MTHFR) C677T polymorphism, and abdominal aortic aneurysm (AAA) remain controversial, with only few studies focused on these associations within the Chinese population. We performed subgroup and interaction analyses in a Chinese Han population to investigate these associations. In all, 155 AAA patients and 310 control subjects were evaluated for serum total homocysteine levels and MTHFR C677T polymorphisms. Multiple logistic regression models were used to evaluate the aforementioned associations. Interaction and stratified analyses were conducted according to age, sex, smoking status, drinking status, and chronic disease histories. The multiple logistic analyses showed a significant association between HHcy and AAA but no significant association between MTHFR C677T polymorphism and AAA. The interaction analysis showed that age and peripheral arterial disease played an interactive role in the association between HHcy and AAA, while drinking status played an interactive role in the association between MTHFR C677T polymorphism and AAA. In conclusion, HHcy is an independent risk factor of AAA in a Chinese Han population, especially in the elderly and peripheral arterial disease subgroups. Longitudinal studies and clinical trials aimed to reduce homocysteine levels are warranted to assess the causal nature of these relationships

Abdominal aortic aneurysm (AAA) is a life-threatening disease that affects up to 9% of men aged >65 years^1^. In Western populations, the mean annual incidence of new AAA diagnosis is 0.4–0.67%^2^,^3^. Rupture of these aneurysms accounts for approximately 8000 annual deaths in the United Kingdom alone and up to 15,000 in the United States^4^,^5^. While AAA and atherosclerosis share several common risk factors, including age, smoking status, hypercholesterolaemia, and hypertension, the strength of the associations between these factors varies between AAA and atherosclerosis^2^. In addition, the inverse associations of diabetes with AAA and aortic diameter suggest a differing pathogenesis for each of the disease processes^6^. Despite the improved understanding of the pathophysiological mechanism and molecular biology of AAA, the aetiology of AAA remains unclear^7^.

Homocysteine (Hcy) is a sulphur-containing non-essential amino acid that functions as a key intermediate during methionine metabolism. Several factors influence Hcy levels, including sex, age, and the enzymes involved in methionine metabolism. A common functional polymorphism in C677T, a gene encoding methylenetetrahydrofolate reductase (MTHFR), is responsible for 70% of the reduction in enzymatic activity^8^,^9^. Elevated Hcy level is known as hyperhomocysteinaemia (HHcy) and has long been suggested as an independent risk factor of coronary heart disease, stroke, and peripheral vascular disease. In addition, previous studies on the MTHFR C677T polymorphism suggested a causal relationship between HHcy and these diseases^10^,^11^. Furthermore, studies that investigated vitamin treatment to reduce Hcy level and the effect of the treatment on cardiovascular or stroke end points showed inconsistent results^12^,^13^,^14^.

Previous studies investigated the association between HHcy and AAA but obtained conflicting results^15^,^16^,^17^,^18^,^19^,^20^,^21^. Whether Hcy level plays a causal role or is simply a bystander in the pathogenic process of AAA remains elusive. Studies have focused on the association between the C677T polymorphism and AAA, while others have reported an association between the T allele and AAA^15^,^17^,^22^,^23^. However, the findings from recent larger case-control studies^24^,^25^ and a cross-sectional study^19^ could not confirm this association. In addition, the interactions of other cardiovascular risk factors such as age, smoking, and hypertension toward these associations are still unknown. Moreover, only few studies^17^ have in fact investigated these associations within a Chinese population. Therefore, the present study aimed to investigate the associations between HHcy, the MTHFR C677T polymorphism, and AAA, by using interaction and stratified analyses to evaluate the influence of different clinical and laboratory features on these associations within the Chinese Han population.

## Methods

### Study population

A case-control study was performed between July 2011 and December 2012 in China^26^. AAA patients (n = 155) and control subjects (n = 310) were enrolled in the study. Patients diagnosed with AAA by using abdominal Doppler ultrasonography or computed tomography (CT) at the Vascular and Endovascular Surgery Department of the Chinese PLA General Hospital in Beijing were enrolled in this study. The control group included non-AAA participants, half (n = 155) of whom were selected from the same hospital (referred to as control group (1) and the other half (n = 155) were healthy subjects selected from communities in Beijing, including urban and suburban districts (referred to as control group (2). The control subjects were sex- and age-matched (within 5 years) with the AAA patients. Participants were excluded based on the following criteria: (1) an ethnic origin other than Han; (2) taking vitamin supplementation, and had renal impairment, malignant tumours, or hypothyroidism); (3) presence of a mental disorder; and (4) pregnancy. This study protocol was approved by the ethics committee of Chinese PLA General Hospital, and informed consent was obtained from all the study participants. All experiments were performed in accordance with relevant guidelines and regulations.

### Data collection procedures

A standardized questionnaire was used to collect data related to the subjects’ demographic characteristics, history of chronic diseases, chronic medication, and lifestyle factors. The participants underwent a standard physical examination. AAA was defined as a maximal infrarenal aortic diameter ≥30 mm. The participants in the control group underwent abdominal ultrasonographic examinations by two trained specialists in order to exclude those with AAA. Biochemical parameters were analysed at the clinical laboratory of the Chinese PLA Hospital. All blood samples were centrifuged for separation of serum within 1 hour of collection and stored at −80 °C until analysis. The total Hcy (tHcy; free and protein bound), total cholesterol, triglyceride, low-density lipoprotein cholesterol, and high-density lipoprotein cholesterol levels were measured by using the Roche Modular chemistry analyser (Roche Diagnostics, Basel, Switzerland). The intra- and inter-assay coefficients of variation were <5% for all of the assays performed. Details of an assessment of major environmental risk factors were reported in our previous study^26^. Subjects were considered to be smokers if they had smoked more than 100 cigarettes over their lifetimes. Drinking was defined as consuming at least 50 mL of white spirits at a time and having a drink at least once a week for a period of >6 months. Self-reported chronic diseases included type 2 diabetes, coronary artery disease (CAD), and ischemic stroke. Hypertension was defined either as a systolic blood pressure (SBP) ≥140 mm Hg or a diastolic blood pressure (DBP) ≥90 mm Hg, or the use of hypotensive drugs. Hyperlipidaemia was defined either as serum concentrations of total cholesterol >5.7 mmol/L, triglyceride >1.7 mmol/L, low-density lipoprotein cholesterol >3.4 mmol/L, or high-density lipoprotein cholesterol <1.0 mmol/L, or the use of hypolipidaemic drugs. Subjects whose ankle brachial indexes were <0.9 were classified as having peripheral arterial disease (PAD). HHcy was defined as a serum tHcy concentration >15 μmol/L, as determined based on the laboratory reference range.

### DNA extraction and genotyping

Genomic DNA was extracted from leukocytes by using the salt fractionation method. DNA samples were stored at −80 °C until analysis. Polymerase chain reaction was performed on a thermal cycler (ABI GeneAmp 9700 384 Dual, ABI, Foster City, CA, USA). Genotypes were analysed with the Typer 4.0 software (MassARRAY Compact System; Sequenom, San Diego, CA, USA).

### Statistical analysis

Data are presented as mean ± standard deviation (SD) or median (interquartile) for continuous variables, and as frequency or percentage for categorical variables. The Mann-Whitney and chi-square tests were used to determine any statistical difference between the means and proportions of the two groups. Multiple logistic regression models were used to evaluate the associations between serum tHcy levels, the MTHFR 677C/T polymorphism, and AAA. Both non-adjusted and multivariate adjusted models (variables adjusted for age, sex, smoking status, drinking status, hypertension, dyslipidaemia, type 2 diabetes, CAD, ischemic stroke, and PAD) were applied. Interaction and stratified analyses were conducted according to age (<70 and ≥ 70 years), smoking status (never smoked and smokers), drinking status (non-drinker and drinkers), and histories of chronic diseases. All of the analyses were performed with the statistical software packages R (http://www.R-project.org, The R Foundation) and EmpowerStats (http://www.empowerstats.com, X&Y Solutions, Inc., Boston, MA). A two-sided significance level of 0.05 was used to evaluate statistical significance.

## Results

### Demographic characteristics

The characteristics of the subjects in the AAA and control groups are presented in Table 1. Satisfactory internal homogeneity was observed among the participants in the two control groups (hospital vs. community population). Therefore, the data from the control group were combined for the analysis. No difference in either sex or age was observed between the AAA and control groups. However, traditional cardiovascular risk factors, including SBP, DBP, and tHcy levels, significantly differed between the AAA and control groups.

### HHcy and Hcy level

**Figure 1:**
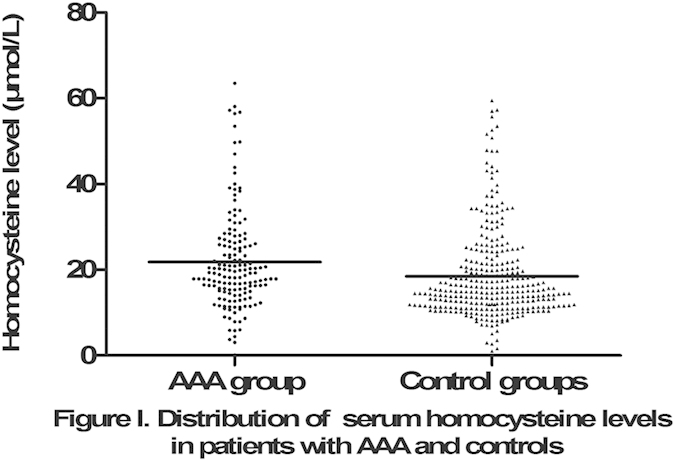
Distribution of serum total homocysteine levels in patients with AAA and control subjects.

HHcy was defined as tHcy levels >15 μmol/L and was detected in 116 (74.80%) of the 155 patients in the AAA group and in 155 (50%) of the 310 subjects in the control group (p < 0.001). The mean serum tHcy level in the AAA group was significantly higher than that in the control group (22.21 ± 13.10 μmol/L vs. 18.49 ± 10.48 μmol/L; Fig. 1).

The serum tHcy levels in the different C677T MTHFR genotypes are presented in Table 2. In patients with AAA, a strong association was observed between the MTHFR genotype and serum tHcy level, as is evident from the fact that the mean serum tHcy levels of the CT or TT genotype carriers were significantly higher than those of the CC genotype carriers (Table 2). In the control group, only the mean serum tHcy levels of the TT genotype carriers were significantly higher than those of the CC genotype carriers (Table 2).

### Relationship between HHcy and AAA

The relationship between HHcy and AAA is presented in Table 3. The univariate logistic analysis revealed a significant association between HHcy and AAA (odds ratio [OR] = 2.97; 95% confidence interval [CI], 1.94−4.55; p < 0.001). Variables were adjusted for age, sex, smoking status, drinking status, hypertension, dyslipidaemia, type 2 diabetes, CAD, ischemic stroke, and PAD. This did not significantly alter the results (OR = 2.84; 95% CI, 1.63–4.93; p < 0.001).

The stratified analyses of the associations between HHcy and AAA are presented in Table 4. Aneurysm size was greater in patients with HHcy than in subjects with normal serum tHcy levels (55.08 ± 16.76 mm vs. 51.53 ± 13.72 mm; p = 0.24), although the difference was not statistically significant. No significant correlation was found between AAA size and serum tHcy levels (*r* = −0.049; p = 0.55).

**Figure 2:**
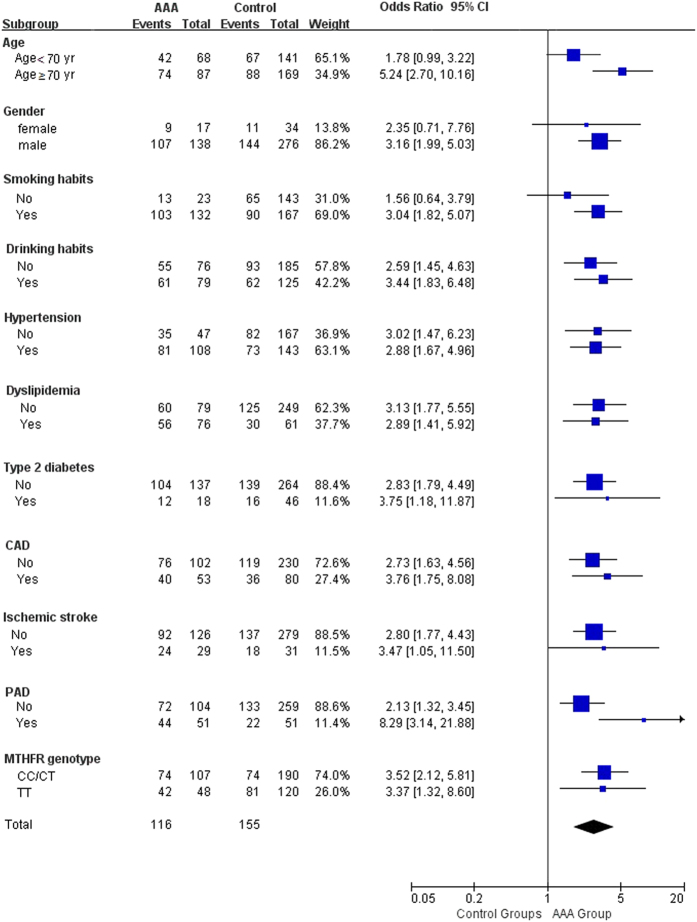
Odds ratios for abdominal aortic aneurysm, according to baseline characteristics.

The interaction analysis revealed that age and PAD played an interactive role in the association between HHcy and AAA (Table 4). The participants aged ≥70 years had higher ORs between HHcy and AAA (OR = 5.24; 95% CI, 2.70–10.16; p < 0.001) than those aged <70 years (OR = 1.78; 95% CI, 0.99–3.22; p = 0.06). In addition, the OR between HHcy and AAA was much higher in the participants with PAD (OR = 8.29; 95% CI, 3.14–21.88; p < 0.001) than in those without a history of PAD (OR = 2.13; 95% CI, 1.32–3.45; p = 0.002; Fig. 2).

### Relationship between the C677T MTHFR genotype and AAA

Genotype distribution and OR, 95% CIs, and p values, as estimated by using logistic regression analysis, for the AAA and control groups are presented in Table 5. The MTHFR677 T allele frequency was similar for both groups, with a value of 0.56 in the AAA group and 0.59 in the control groups (p = 0.37; Table 5). The frequencies of the CC, CT, and TT genotypes in the AAA group were 19.4%, 49.7%, and 31%, respectively. The corresponding frequencies in the combined control groups were 21%, 40.3%, and 38.7%, respectively (Table 5). The univariate logistic analysis revealed no association between the C677T MTHFR genotype and AAA (CT vs. CC: OR = 1.33; 95% CI, 0.80–2.24; p = 0.05; TT vs. CC: OR, 0.87; 95% CI, 0.50–1.50; p = 0.61). Variables were adjusted for age, sex, smoking status, drinking status, hypertension, dyslipidaemia, type 2 diabetes, CAD, ischemic stroke, and PAD. This did not alter the results (Table 5).

The results of the stratified analyses of the association between the C677T MTHFR genotype and the clinical and laboratory results are presented in Table 6. The aneurysm size was greater in the patients with the CT or TT genotype than in those with the CC genotype (55.11 ± 16.75 mm vs. 50.33 ± 12.43 mm; p = 0.24), although the difference was not statistically significant. AAA size was not significantly correlated with the C677T MTHFR genotype (*r* = 0.0392; p = 0.63).

Interaction analysis revealed that drinking habits influenced the association between the MTHFR C677T polymorphism and AAA (Table 6). The OR between the MTHFR C677T polymorphism and AAA was higher in those who consumed alcohol (OR = 2.46; 95% CI, 1.10–5.50; p = 0.03) than in those who did not drink (OR = 0.61; 95% CI, 0.33–1.14; p = 0.12; Table 6).

## Discussion

In the present study, HHcy was identified as a significant and independent risk factor of AAA in the Chinese Han population. Patients with HHcy had a 2.84-fold greater risk of AAA than subjects with normal tHcy levels. Indeed, a 1 μmol/L increase in tHcy level was associated with a 2% higher risk of AAA, further confirming the relationship between HHcy and AAA. Moreover, we found that both age and PAD significantly influenced this relationship, suggesting that these two factors may interact with the Hcy biological pathway to stimulate the development of AAA. We were unable to confirm the association between the MTHFR C677T polymorphism and AAA susceptibility. Furthermore, we found no significant correlation between AAA size, MTHFR C677T polymorphism, and serum tHcy level. However, we found that alcohol consumption influenced the association between the MTHFR C677T polymorphism and AAA. This was apparent from the fact that subjects with the MTHFR TT or CT genotypes in conjunction with alcohol consumption had a 2.46-fold significantly higher risk of AAA than those with the MTHFR CC genotype. No such associations were observed in subjects who did not consume alcohol, suggesting that drinking habits may play a role in the relationship between MTHFR C677T polymorphism and AAA.

Previous studies that investigated the relationship between HHcy and AAA reported inconsistent results. In a case-control study, Brunelli *et al*.^16^ originally showed that AAA risk increased by up to sixfold in the presence of HHcy and that this association was further observed in a subgroup without evidence of atherosclerosis. Six subsequent case-control studies^15^,^17^,^18^,^19^,^22^,^27^ reported similar results, with an OR ranging from 1.10 to 36.83, further supporting the association between HHcy and AAA. Wong *et al*.^19^ conducted the largest epidemiological study yet with 4248 men and confirmed the independent association between HHcy and AAA (OR = 1.45; 95% CI, 1.10–1.91). However, Peeters *et al*.^20^ showed that low vitamin B6 level, as opposed to plasma Hcy level, was in fact an independent risk factor of AAA and recommended that future studies that investigate the association between HHcy and AAA should consider confounding factors and subgroup analyses.

In our study, the proportion of subjects with HHcy was significantly higher in the AAA group than in the control groups. In addition, the mean serum tHcy level was higher in the AAA group than in the control groups. The multivariate analysis revealed that the risk of AAA was almost threefold in the presence of HHcy (OR = 2.84; 95% CI, 1.63–4.93). In a recent study^17^, Cao *et al*. confirmed the association between HHcy and AAA in a Chinese population. When compared with the present study, although the OR of AAA in association with HHcy was similar at 2.84, the tHcy level, HHcy prevalence, and the relationship between Hcy and AAA size varied widely. Thus, further studies are needed particularly in the Chinese population in order to confirm this association.

Whether circulating tHcy levels play a role in predicting progression to AAA is controversial. Brunelli *et al*.^16^ observed that aneurysm size was significantly larger in patients with HHcy than in those with normal Hcy levels. Subsequent studies^15^,^17^ confirmed that patients with HHcy had a significantly larger aneurysm diameter than those with normal tHcy levels. Sofi *et al*.^15^ observed that abdominal aortic diameter was significantly correlated with Hcy level (*r* = 0.13; p = 0.005). Furthermore, metaregression analyses^28^ revealed that the larger the diagnosed diameter, the greater the risk of AAA in subjects with HHcy. In addition, a positive dose-response relationship between tHcy level and abdominal aortic diameter was recently reported^19^. The report indicated that every 5 μmol/L increment in tHcy level was associated with a 0.15mm (95% CI, 0.01–0.28 mm) larger mean aortic diameter. Moreover, Halazun *et al*.^29^ showed a correlation between Hcy level and the growth rate of AAA, where expansion rates (and a consequent increased risk of rupture) were observed to be significantly higher in patients with HHcy than in subjects with normal tHcy levels. However, three additional studies^15^,^18^,^27^ reported conflicting results and did not find a significantly larger AAA diameter in patients with HHcy. Moreover, Lindqvist *et al*.^21^ showed that B12 levels, as opposed to Hcy levels, had a significant inverse correlation to aneurysm diameter. Finally, yet another cross-sectional study^30^ showed no significant association between tHcy level and abdominal aortic diameter.

In our study, the aneurysm diameter was not significantly greater in patients with HHcy than in subjects with normal tHcy levels. In addition, we found no correlation between tHcy level and aneurysm diameter, suggesting that the present study may not have been adequate for evaluating the influence of Hcy level on the progression of AAA, especially as it was not a cohort study. We therefore recommend that future studies should follow-up AAA patients for several years in order to provide a more accurate evaluation of the relationship between HHcy and AAA progression.

All three previously published meta-analyses^28^,^31^,^32^ have confirmed that HHcy is a risk factor of AAA. However, the high heterogeneity in these studies weakened the level of evidence provided, providing further confirmation that future studies should consider additional factors such as participant selection, method of tHcy measurement, and subgroup analysis. Our subgroup analysis revealed that in the participants aged ≥70 years, the odds of experiencing an AAA in conjunction with HHcy was 5.24 (95% CI, 2.70–10.16) when compared with those with normal tHcy concentrations. In participants aged <70 years, HHcy did not significantly increase the risk of AAA (OR = 1.78; 95% CI, 0.99–3.22). Our results also showed that patients with PAD had higher odds of having AAA with HHcy than participants without a history of PAD. Indeed, these findings indicate that age and PAD may interact with the Hcy biological pathway and potentially stimulate AAA development. Thus, future studies on this topic should consider both factors.

Whether Hcy level plays a direct causal role in the pathogenesis of AAA or is merely an innocent bystander remains elusive. Hcy level influences a wide range of molecular pathways that could be of relevance to the pathogenesis of AAA, including endothelial dysfunction^33^,^34^,^35^, proteolysis^36^, and inflammation^37^,^38^. A recent animal study by Liu *et al*.^32^ demonstrated that HHcy contributes to the development of AAA through a range of molecular pathways, including endothelial dysfunction, matrix remodelling, and inflammatory and immune reactions, a process that could be reversible with folic acid supplementation. However, Arapoglou *et al*.^39^ tested aneurysm specimens from 89 male patients and showed that tHcy levels were not associated with high-grade tissue inflammation (OR = 0.9; 95% CI, 0.9–1.02), providing evidence against a major effect of tHcy level on AAA tissue inflammation.

To overcome the problem of reverse causality, several studies have assessed the MTHFR genotype in patients with AAA. However, the association between the MTHFR genotype and AAA is still controversial. Five case-control studies^15^,^16^,^17^,^22^,^23^ and three meta-analyses^25^,^40^,^41^ showed that carriers of the MTHFR 677T allele had an increased risk of AAA. Brunelli *et al*.^16^ were the first to suggest that patients with AAA were more likely to be homozygous for the C677T variant of the MTHFR gene than the control group. Strauss *et al*.^22^, along with three other case-control studies^15^,^17^,^23^, further confirmed this genotypic association. In the study of Strauss *et al*.^22^, patients who were heterozygous for the mutation had a 4.4-fold higher risk of AAA than those who were homozygous for the C allele. However, it should be noted that study sample in that study was small and that the control group was not well matched. Sofi *et al*.^15^ and Ferrara *et al*.^23^ both showed a higher T-allele frequency in AAA patients than in control groups. A recent study^17^ in a Chinese population showed that the frequency of the homozygous (TT) genotype was significantly higher in patients with AAA than in controls and reported a risk estimate of 1.79 for AAA with the MTHFR 677TT genotype (95% CI, 1.24–2.58). However, five additional case-control studies^19^,^20^,^24^,^25^,^42^ showed no significant association between the MTHFR 677T allele and AAA. Both Peeters *et al*.^20^ and Saratzis *et al*.^25^ (a Greek cohort study) showed only a modest relationship between the MTHFR 677TT genotype and the occurrence of AAA, with no statistically significant difference. Giusti *et al*.^24^, Wong *et al*.^19^, and Saratzis *et al*.^25^ (a UK cohort study) observed a modest negative association between the MTHFR 677TT genotype and the occurrence of AAA, but the association was not statistically significant. Studies^15^,^19^,^22^,^42^ have shown that the aneurysm diameter was no larger in the AAA patients with the MTHFR 677TT genotype than in those with the CC MTHFR genotype. Wong *et al*.^19^ found no association between the T allele and the presence of AAA and aortic diameter, and further deduced that a study would require more than 30,000 men with the TT genotype in order to detect an effect size of a 0.03-mm increase in mean aortic diameter in association with a 1 mmol/L increment in tHcy level. In stark contrast to the results reported by Cao *et al*.^31^ in a Chinese population, our study results show no association between the C677T MTHFR genotype and AAA (CT vs. CC, OR = 0.95; 95% CI, 0.50–1.83, p = 0.88; TT vs. CC, OR = 0.72, 95% CI, 0.36–1.42, p = 0.34). Furthermore, we found no significant association between the MTHFR C677T allele and aneurysm diameter. In addition, we found no significantly higher increase in aneurysm size in patients with the CT or TT genotype than in those with the CC genotype, which suggests that our study may not have been adequate for evaluating the influence of MTHFR C677T polymorphism on AAA.

While a meta-analysis may confirm the association between the MTHFR genotype and AAA, the possibility of high sample heterogeneity and publication bias should be considered. For example, in a meta-analysis by Saratzis *et al*.^25^, if the results of the study by Cao *et al*.^17^ are included together with our own, the reported significant risk estimate of 1.07 (95% CI, 1.02–1.12) for AAA with the MTHFR 677T genotype increases to a non-significant risk estimate of 1.13 (95% CI, 0.99–1.29). Therefore, more-comprehensive meta-analyses are needed and future studies should consider confounding factors and their interactions, as well as subgroup analyses. Ferrara *et al*.^23^ divided the patients in the AAA group into two groups according to age and found that the frequency of the 677T allele in younger AAA patients was significantly higher than that in older AAA patients, a result we were not able to confirm in the present study. However, in our study, among the patients who consumed alcohol, the number of carriers of the MTHFR 677T allele and the risk of AAA was higher (OR = 2.46; 95% CI, 1.10–5.50; p = 0.03) than among the participants who did not drink (OR = 0.61; 95% CI, 0.33–1.14; p = 0.12). This suggests that drinking may play an important role in the association between MTHFR C677T polymorphism and AAA.

Our study provides evidence of the associations between serum tHcy level, MTHFR C677T polymorphism, and AAA in a Chinese Han population by performing subgroup and interaction analyses. When interpreting our findings, several limitations in the present study should be considered. First, a case-control study design provides a challenge in terms of determining whether the association between HHcy and AAA is causal or not. Second, information regarding the beneficial effect of vitamin status is lacking; therefore, we did not evaluate the roles of vitamins in the association between HHcy and AAA. Third, self-reported histories of chronic diseases might have caused a recall bias and affected the accuracy of the estimation of disease prevalence. These should be considered and included in future studies.

In conclusion, HHcy is an independent risk factor of AAA in a Chinese Han population, particularly in the elderly and smoking subgroups. The association between the MTHFR C677T polymorphism and AAA observed in drinking subjects may indicate a causal link between HHcy and AAA, despite the small sample size. Longitudinal studies and clinical trials of folic acid for reducing Hcy levels are warranted to assess the causal nature of these relationships.

## Additional Information

**How to cite this article**: Liu, J. *et al*. Hyperhomocysteinaemia is an independent risk factor of abdominal aortic aneurysm in a Chinese Han population. *Sci. Rep.* **6**, 17966; doi: 10.1038/srep17966 (2016).

## Figures and Tables

**Table 1:** Demographic characteristics of the abdominal aortic aneurysm (AAA) group and the control groups.

		Control groups (n = 310)			
Variable	AAA group (n = 155)	Control group 1 (n = 155)	Control group 2 (n = 155)	P 1 values	P 2 values
Age, years	69.18 ± 9.94	69.59 ± 10.86	69.45 ± 8.95	0.89	0.85
Sex, male	138 (89.0)	138 (89.0)	138 (89.0)	1.00	1.00
Smoking habits	132 (85.2)	69 (44.5)	98 (63.2)	<0.01	<0.01
Drinking habits	79 (51.0)	58 (37.4)	67 (43.2)	0.30	0.03
SBP	130.60 ± 17.32	136.70 ± 17.78	137.53 ± 21.04	0.59	0.003
DBP	78.66 ± 10.71	80.22 ± 10.40	87.07 ± 12.73	<0.001	<0.001
Hypertension	108 (69.7)	64 (41.3)	79 (51.0)	0.09	<0.001
Dyslipidaemia	118 (76.1)	65 (41.9)	73 (47.1)	0.36	<0.01
Type 2 diabetes	18 (11.6)	27 (17.4)	19 (12.3)	0.20	0.34
CAD	53 (34.2)	36 (23.2)	44 (28.4)	0.30	0.06
Ischemic stroke	29 (18.7)	17 (11.0)	14 (9.0)	0.57	0.01
PAD	51 (32.9)	22 (14.2)	29 (18.7)	0.28	<0.01
Aorta diameter, mm	52.00(44.00-61.00)	17.00(15.40-20.00)	16.10(14.62-17.70)	0.10	<0.001
tHcy, μmol/l	22.21 ± 13.10	18.37 ± 9.61	18.60 ± 11.32	0.10	<0.001

CAD, Coronary artery disease; PAD, peripheral arterial disease; tHcy, serum total homocysteine.

Continuous data are shown as the mean ± standard deviation or median (lower quantile, upper quantile), and categorical data as number (%).

P1 values were calculated by comparing demographic or clinical characteristics between two control groups and P2 values were calculated by comparing characteristics between AAA and all controls.

**Table 2:** Serum homocysteine levels of different C677T MTHFR genotype in the abdominal aortic aneurysm (AAA) group and the control groups.

		Homocysteine (μmol/L)
AAA group	CC	14.70 ± 6.58
	CT	20.98 ± 10.93^*^
	TT	28.88 ± 16.07^**^
Control groups	CC	14.56 ± 6.99
	CT	15.79 ± 7.72
	TT	23.43 ± 12.50^**^

*CT vs CC: p < .05; **TT vs CT and TT vs CC: p < .05.

**Table 3:** Multinomial logistic regression models evaluating the association of serum total homocysteine (tHcy) with abdominal aortic aneurysm (AAA).

Variable	Crude	P	Adjusted^a^	P
	OR, 95% CI		OR, 95% CI	
Hcy, 1 μmol/L	1.03 (1.01, 1.04)	<0.001	1.02 (1.00, 1.05)	0.03
Hcy < 15 μmol/L	Ref	—	Ref	—
Hcy ≥ 15 μmol/L	2.97 (1.94, 4.55)	<0.001	2.84 (1.63, 4.93)	<0.001

^a^Adjusted for age, sex, smoking status, drinking status, hypertension, dyslipidaemia, type 2 diabetes, coronary artery disease (CAD), ischemic stroke, and peripheral arterial disease (PAD).

CI, confidence interval; OR, odds ratio.

**Table 4:** Association between hyperhomocystenaemia (HHcy) and abdominal aortic aneurysm (AAA) according to baseline characteristics.

Subgroup	AAA group(n)		Control groups(n)		OR, 95% CI	P Value	P Value forInteraction
	HHcy	nHcy	HHcy	nHcy			
Age							0.016
<70 yr	42	26	67	74	1.78 (0.99, 3.22)	0.06	
≥ 70 yr	74	13	88	81	5.24 (2.70, 10.16)	<0.001	
Sex							0.65
Male	107	31	144	132	3.16 (1.99, 5.03)	<0.001	
Female	9	8	11	23	2.35 (0.713, 7.76)	0.160	
Smoking Habits							0.20
No	13	10	65	78	1.56 (0.642, 3.79)	0.326	
Yes	103	29	90	77	3.04 (1.82, 5.07)	<0.001	
Drinking Habits							0.51
No	55	21	93	92	2.59 (1.45, 4.63)	0.001	
Yes	61	18	62	63	3.44 (1.83, 6.48)	<0.001	
Hypertension							0.92
No	35	12	82	85	3.02 (1.47, 6.23)	0.003	
Yes	81	27	73	70	2.88 (1.67, 4.96)	<0.001	
Dyslipidaemia							0.88
No	60	19	125	124	3.13 (1.77, 5.55)	<0.001	
Yes	56	20	30	31	2.89 (1.41, 5.92)	0.004	
Type 2 diabetes							0.66
No	104	33	139	125	2.83 (1.79, 4.49)	<0.001	
Yes	12	6	16	30	3.75 (1.18, 11.87)	0.025	
CAD							0.49
No	76	26	119	111	2.73 (1.63, 4.56)	<0.001	
Yes	40	13	36	44	3.76 (1.75, 8.08)	<0.001	
Ischemic stroke							0.74
No	92	34	137	142	2.80 (1.77, 4.43)	<0.001	
Yes	24	5	18	13	3.47 (1.05, 11.50)	0.042	
PAD							0.01
No	72	32	133	126	2.13 (1.32, 3.45)	0.002	
Yes	44	7	22	29	8.29 (3.14, 21.88)	<0.001	
MTHFR genotype							0.94
CC/CT	74	33	74	116	3.52 (2.12, 5.81)	<0.001	
TT	42	6	81	39	3.37 (1.32, 8.60)	0.011	

OR, odds ratio; CI, confidence interval.

CAD, Coronary artery disease; PAD, peripheral arterial disease; nHcy, normal serum homocysteine level; MTHFR, methylenetetrahydrofolate reductase.

**Table 5:** Allele and genotype frequencies of MTHFR C677T polymorphism in patients and controls.

C677T MTHFR Genotype	AAA, No. (%)	Control, No. (%)	Crude	P	Adjusted^a^	P
			OR (95% CI)		OR (95% CI)	
CC	30 (19.4)	65 (21.0)	1.0 (referent)	—	1.0 (referent)	—
CT	77 (49.7)	125 (40.3)	1.33 (0.80, 2.24)	0.27	0.95 (0.496, 1.83)	0.88
TT	48 (31.0)	120 (38.7)	0.87 (0.50, 1.50)	0.61	0.72 (0.36, 1.42)	0.34
TT/CT	125 (80.6)	245 (79.0)	1.11 (0.68, 1.79)	0.68	0.84 (0.46, 1.54)	0.57

^a^Adjusted for age, sex, smoking status, drinking status, hypertension, dyslipidaemia, type 2 diabetes, coronary artery disease (CAD), ischemic stroke, and peripheral arterial disease (PAD).

CI, Confidence interval; OR, odds ratio; MTHFR, methylenetetrahydrofolate reductase.

**Table 6:** Association between C677T MTHFR genotype and AAA according to baseline characteristics.

Subgroup	AAA group (n)	Control groups (n)		CC	OR, 95% CI	P Value	P Value for Interaction
	TT/CT	CC	TT/CT				
Age							0.06
<70 yr	50	18	113	28	0.69 (0.35, 1.36)	0.28	
≥70 yr	75	12	132	37	1.75 (0.86, 3.56)	0.12	
Sex							0.08
Male	113	25	215	61	1.28 (0.76, 2.15)	0.35	
Female	12	5	30	4	0.32 (0.073, 1.40)	0.13	
Smoking Habits							0.12
No	15	8	112	31	0.52 (0.20, 1.34)	0.17	
Yes	110	22	133	34	1.28 (0.71, 2.31)	0.42	
Drinking Habits							0.006
No	55	21	150	35	0.61 (0.33, 1.14)	0.12	
Yes	70	9	95	30	2.46 (1.10, 5.50)	0.03	
Hypertension							0.11
No	36	11	138	29	0.69 (0.31, 1.51)	0.35	
Yes	89	19	107	36	1.58 (0.85, 2.94)	0.15	
Dyslipidaemia							0.23
No	60	19	197	52	0.83 (0.46, 1.52)	0.55	
Yes	65	11	48	13	1.60 (0.66, 3.88)	0.30	
Type 2 diabetes							0.46
No	109	28	208	56	1.05 (0.63, 1.74)	0.86	
Yes	16	2	37	9	1.95 (0.38, 10.04)	0.43	
CAD							0.60
No	81	21	177	53	1.15 (0.65, 2.04)	0.62	
Yes	44	9	68	12	0.86 (0.34, 2.22)	0.76	
Ischemic stroke							0.93
No	101	25	220	59	1.08 (0.64, 1.83)	0.76	
Yes	24	5	25	6	1.15 (0.31, 4.28)	0.83	
PAD							0.46
No	84	20	208	51	1.03 (0.58, 1.83)	0.92	
Yes	41	10	37	14	1.55 (0.62, 3.91)	0.35	
Hcy							0.19
HHcy	101	15	132	23	1.17 (0.58, 2.36)	0.66	
nHcy	24	15	113	42	0.60 (0.29, 1.24)	0.17	

CI, confidence interval; OR, odds ratio.

MTHFR, methylenetetrahydrofolate reductase; CAD, coronary artery disease; PAD, peripheral arterial disease; HHcy, hyperhomocysteinaemia ; nHcy, normal serum homocysteine level.

## References

[b1] Thompson R. W. Detection and management of small aortic aneurysms. N Engl J Med 346, 1484–6 (2002). 12000820 10.1056/NEJM200205093461910

[b2] Forsdahl S. H., Singh K., Solberg S. & Jacobsen B. K. Risk factors for abdominal aortic aneurysms: a 7-year prospective study: the Tromso Study, 1994–2001. Circulation 119, 2202–8 (2009). 19364978 10.1161/CIRCULATIONAHA.108.817619

[b3] Lederle F. A. . Yield of repeated screening for abdominal aortic aneurysm after a 4-year interval. Aneurysm Detection and Management Veterans Affairs Cooperative Study Investigators. Arch Intern Med 160, 1117–21 (2000). 10789604 10.1001/archinte.160.8.1117

[b4] Gillum R. F. Epidemiology of aortic aneurysm in the United States. J Clin Epidemiol 48, 1289–98 (1995). 7490591 10.1016/0895-4356(95)00045-3

[b5] Thompson M. M. Controlling the expansion of abdominal aortic aneurysms. Br J Surg 90, 897–8 (2003). 12905540 10.1002/bjs.4280

[b6] Le M. T., Jamrozik K., Davis T. M. & Norman P. E. Negative association between infra-renal aortic diameter and glycaemia: the Health in Men Study. Eur J Vasc Endovasc Surg 33, 599–604 (2007). 17307366 10.1016/j.ejvs.2006.12.017

[b7] Weintraub N. L. Understanding abdominal aortic aneurysm. N Engl J Med 361, 1114–6 (2009). 19741234 10.1056/NEJMcibr0905244 PMC3791612

[b8] Refsum H. . Facts and recommendations about total homocysteine determinations: an expert opinion. Clin Chem 50, 3–32 (2004). 14709635 10.1373/clinchem.2003.021634

[b9] Bathum L. . Genetic and environmental influences on plasma homocysteine: results from a Danish twin study. Clin Chem 53, 971–9 (2007). 17412799 10.1373/clinchem.2006.082149

[b10] Wald D. S., Law M. & Morris J. K. Homocysteine and cardiovascular disease: evidence on causality from a meta-analysis. BMJ 325, 1202 (2002). 12446535 10.1136/bmj.325.7374.1202 PMC135491

[b11] Bautista L. E., Arenas I. A., Penuela A. & Martinez L. X. Total plasma homocysteine level and risk of cardiovascular disease: a meta-analysis of prospective cohort studies. J Clin Epidemiol 55, 882–7 (2002). 12393075 10.1016/s0895-4356(02)00434-1

[b12] Bonaa K. H. . Homocysteine lowering and cardiovascular events after acute myocardial infarction. N Engl J Med 354, 1578–88 (2006). 16531614 10.1056/NEJMoa055227

[b13] Qin X. . Homocysteine-lowering therapy with folic acid is effective in cardiovascular disease prevention in patients with kidney disease: a meta-analysis of randomized controlled trials. Clin Nutr 32, 722–7 (2013). 23313356 10.1016/j.clnu.2012.12.009

[b14] Huo Y. . Efficacy of folic acid therapy in primary prevention of stroke among adults with hypertension in China: the CSPPT randomized clinical trial. JAMA 313, 1325–35 (2015). 25771069 10.1001/jama.2015.2274

[b15] Sofi F. . High levels of homocysteine, lipoprotein (a) and plasminogen activator inhibitor-1 are present in patients with abdominal aortic aneurysm. Thromb Haemost 94, 1094–8 (2005). 16363254

[b16] Brunelli T. . High prevalence of mild hyperhomocysteinemia in patients with abdominal aortic aneurysm. J Vasc Surg 32, 531–6 (2000). 10957660 10.1067/mva.2000.107563

[b17] Cao H. . Hyperhomocysteinaemia, low folate concentrations and MTHFR C677T mutation in abdominal aortic aneurysm. Vasa 43, 181–8 (2014). 24797049 10.1024/0301-1526/a000347

[b18] Spark J. I., Laws P. & Fitridge R. The incidence of hyperhomocysteinaemia in vascular patients. Eur J Vasc Endovasc Surg 26, 558–61 (2003). 14532886 10.1016/s1078-5884(03)00381-2

[b19] Wong Y. Y. . Plasma total homocysteine is associated with abdominal aortic aneurysm and aortic diameter in older men. J Vasc Surg 58, 364–70 (2013). 23643559 10.1016/j.jvs.2013.01.046

[b20] Peeters A. C. . Low vitamin B6, and not plasma homocysteine concentration, as risk factor for abdominal aortic aneurysm: a retrospective case-control study. J Vasc Surg 45, 701–5 (2007). 17398378 10.1016/j.jvs.2006.12.019

[b21] Lindqvist M., Hellstrom A. & Henriksson A. E. Abdominal aortic aneurysm and the association with serum levels of Homocysteine, vitamins B6, B12 and Folate. Am J Cardiovasc Dis 2, 318–22 (2012). 23173106 PMC3499938

[b22] Strauss E., Waliszewski K., Gabriel M., Zapalski S. & Pawlak A. L. Increased risk of the abdominal aortic aneurysm in carriers of the MTHFR 677T allele. J Appl Genet 44, 85–93 (2003). 12590185

[b23] Ferrara F. . Methylenetetrahydrofolate reductase mutation in subjects with abdominal aortic aneurysm subdivided for age. Clin Hemorheol Microcirc 34, 421–6 (2006). 16614466

[b24] Giusti B. . Genetic analysis of 56 polymorphisms in 17 genes involved in methionine metabolism in patients with abdominal aortic aneurysm. J Med Genet 45, 721–30 (2008). 18635682 10.1136/jmg.2008.057851

[b25] Saratzis A. . Association between seven single nucleotide polymorphisms involved in inflammation and proteolysis and abdominal aortic aneurysm. J Vasc Surg 61, 1120–8 e1 (2015). 24613192 10.1016/j.jvs.2013.11.099

[b26] Wei Y. . Association of polymorphisms on chromosome 9p21.3 region with increased susceptibility of abdominal aortic aneurysm in a Chinese Han population. J Vasc Surg 59, 879–85 (2014). 24365123 10.1016/j.jvs.2013.10.095

[b27] Warsi A. A., Davies B., Morris-Stiff G., Hullin D. & Lewis M. H. Abdominal aortic aneurysm and its correlation to plasma homocysteine, and vitamins. Eur J Vasc Endovasc Surg 27, 75–9 (2004). 14652841 10.1016/j.ejvs.2003.09.001

[b28] Takagi H., Umemoto T. & Group A. A meta-analysis of circulating homocysteine levels in subjects with versus without abdominal aortic aneurysm. Int Angiol 34, 229–37 (2015). 24732583

[b29] Halazun K. J. . Hyperhomocysteinaemia is associated with the rate of abdominal aortic aneurysm expansion. Eur J Vasc Endovasc Surg 33, 391–4; discussion 395-6 (2007). 17164089 10.1016/j.ejvs.2006.10.022

[b30] Laughlin G. A. . Abdominal aortic diameter and vascular atherosclerosis: the Multi-Ethnic Study of Atherosclerosis. Eur J Vasc Endovasc Surg 41, 481–7 (2011). 21236707 10.1016/j.ejvs.2010.12.015 PMC3070051

[b31] Cao H. . Homocysteine level and risk of abdominal aortic aneurysm: a meta-analysis. PLoS One 9, e85831 (2014). 24465733 10.1371/journal.pone.0085831 PMC3897527

[b32] Liu Z. . Hyperhomocysteinemia Exaggerates Adventitial Inflammation and Angiotensin II-Induced Abdominal Aortic Aneurysm in Mice. Circ Res 111, 1261–73 (2012). 22912384 10.1161/CIRCRESAHA.112.270520

[b33] Abahji T. . Acute hyperhomocysteinaemia after oral methionine stress leads to macro- and microvascular endothelial dysfunction. Medizinische Klinik 101, 863–863 (2006).

[b34] Chambers J. C., McGregor A., Jean-Marie J. & Kooner J. S. Acute hyperhomocysteinaemia and endothelial dysfunction. Lancet 351, 36–37 (1998). 9433433 10.1016/S0140-6736(05)78090-9

[b35] San Cheang W. . Black tea protects against hypertension-associated endothelial dysfunction through alleviation of endoplasmic reticulum stress. Sci Rep 5, 10340 (2015). 25976123 10.1038/srep10340 PMC4432571

[b36] Charpiot P. . Hyperhomocysteinemia induces elastolysis in minipig arteries: Structural consequences, arterial site specificity and effect of captopril-hydrochlorothiazide. Matrix Biology 17, 559–574 (1998). 9923650 10.1016/s0945-053x(98)90108-1

[b37] Zhang Q., Zeng X., Guo J. & Wang X. Oxidant stress mechanism of homocysteine potentiating Con A-induced proliferation in murine splenic T lymphocytes. Cardiovasc Res 53, 1035–42 (2002). 11922914 10.1016/s0008-6363(01)00541-7

[b38] Zhang D. . Severe hyperhomocysteinemia promotes bone marrow-derived and resident inflammatory monocyte differentiation and atherosclerosis in LDLr/CBS-deficient mice. Circ Res 111, 37–49 (2012). 22628578 10.1161/CIRCRESAHA.112.269472 PMC3412115

[b39] Arapoglou V. . The influence of total plasma homocysteine and traditional atherosclerotic risk factors on degree of abdominal aortic aneurysm tissue inflammation. Vasc Endovascular Surg 43, 473–9 (2009). 19640909 10.1177/1538574409334345

[b40] Thompson A. R., Drenos F., Hafez H. & Humphries S. E. Candidate gene association studies in abdominal aortic aneurysm disease: a review and meta-analysis. Eur J Vasc Endovasc Surg 35, 19–30 (2008). 17920311 10.1016/j.ejvs.2007.07.022

[b41] McColgan P., Peck G. E., Greenhalgh R. M. & Sharma P. The genetics of abdominal aortic aneurysms: a comprehensive meta-analysis involving eight candidate genes in over 16,700 patients. Int Surg 94, 350–8 (2009). 20302034

[b42] Jones G. T., Harris E. L., Phillips L. V. & van Rij A. M. The methylenetetrahydrofolate reductase C677T polymorphism does not associate with susceptibility to abdominal aortic aneurysm. European Journal of Vascular and Endovascular Surgery 30, 137–142 (2005). 15996600 10.1016/j.ejvs.2005.02.047

